# Peposertib, a DNA-PK Inhibitor, Enhances the Anti-Tumor Efficacy of Topoisomerase II Inhibitors in Triple-Negative Breast Cancer Models

**DOI:** 10.3390/ijms25105120

**Published:** 2024-05-08

**Authors:** Steffie Revia, Felix Neumann, Julia Jabs, Florian Orio, Christian Sirrenberg, Astrid Zimmermann, Christiane Amendt, Joachim Albers

**Affiliations:** Research Unit Oncology, Merck Healthcare KGaA, Darmstadt, Germany; steffie.revia@merckgroup.com (S.R.);

**Keywords:** TNBC, DNA repair, NHEJ, DNA-PK, peposertib

## Abstract

Triple-negative breast cancer (TNBC) remains the most lethal subtype of breast cancer, characterized by poor response rates to current chemotherapies and a lack of additional effective treatment options. While approximately 30% of patients respond well to anthracycline- and taxane-based standard-of-care chemotherapy regimens, the majority of patients experience limited improvements in clinical outcomes, highlighting the critical need for strategies to enhance the effectiveness of anthracycline/taxane-based chemotherapy in TNBC. In this study, we report on the potential of a DNA-PK inhibitor, peposertib, to improve the effectiveness of topoisomerase II (TOPO II) inhibitors, particularly anthracyclines, in TNBC. Our in vitro studies demonstrate the synergistic antiproliferative activity of peposertib in combination with doxorubicin, epirubicin and etoposide in multiple TNBC cell lines. Downstream analysis revealed the induction of ATM-dependent compensatory signaling and p53 pathway activation under combination treatment. These in vitro findings were substantiated by pronounced anti-tumor effects observed in mice bearing subcutaneously implanted tumors. We established a well-tolerated preclinical treatment regimen combining peposertib with pegylated liposomal doxorubicin (PLD) and demonstrated strong anti-tumor efficacy in cell-line-derived and patient-derived TNBC xenograft models in vivo. Taken together, our findings provide evidence that co-treatment with peposertib has the potential to enhance the efficacy of anthracycline/TOPO II-based chemotherapies, and it provides a promising strategy to improve treatment outcomes for TNBC patients.

## 1. Introduction

Triple-negative breast cancer (TNBC) is an aggressive subtype of breast cancer, with a poor prognosis and limited treatment options. Unlike other subtypes of breast cancer, TNBC lacks the expression of estrogen receptor (ER), progesterone receptor (PR) and human epidermal growth factor receptor 2 (HER2), making it particularly challenging to treat. TNBC accounts for 10–20% of all breast cancer cases and is associated with a higher rate of recurrence, an increased risk of distant metastasis and a poor prognosis compared to other subtypes of breast cancer [1,2]. Currently, there are no effective targeted therapies available for the treatment of TNBC, leaving chemotherapy regimens that incorporate anthracycline- and taxane-based therapeutics as the standard of care (SoC). Approximately 30% of patients with TNBC respond well to anthracycline- or taxane-based SoC chemotherapy regimens, but the remaining patients demonstrate limited improvements in clinical outcomes, highlighting the critical need for effective strategies to improve chemotherapy responses [3,4]. Anthracyclines such as doxorubicin and epirubicin exert their cytotoxic effects by inhibiting type II topoisomerase (Topo II) enzymes, which are essential for DNA replication and transcription [5]. The mechanism of action of Topo II inhibitors involves the stabilization of the cleavage complex formed by Topo II and DNA, preventing the enzyme from re-ligating the break and releasing the DNA [6]. The stabilized cleavage complex then becomes a cytotoxic intermediate, ultimately leading to DNA double-strand breaks (DSBs) [7]. In mammalian cells, the nonhomologous-end-joining (NHEJ) repair pathway with DNA-dependent protein kinase (DNA-PK) plays a key role in this process and is critical for repairing Topo II-mediated DNA damage [6]. DNA-PK is a serine/threonine kinase and works in co-ordination with five additional factors, Ku70, Ku80, XRCC4, ligase IV and Artemis, to repair DNA DSBs [8]. The discovery of the key role of DNA-PK in DNA damage repair highlighted the potential of DNA-PK inhibitors, such as peposertib, to block DNA repair and, therefore, enhance the efficacy of DNA-damaging agents [8]. Peposertib is a potent, selective and orally bioavailable DNA-PK inhibitor, which, in both preclinical models and clinical trials, strongly potentiates the anti-tumor effects of ionizing radiation and DNA DSB-inducing agents, including anthracyclines [8,9,10]. The combination potential of peposertib with various DNA-damaging treatment modalities, including pegylated liposomal doxorubicin (PLD), is currently being evaluated in early-stage clinical studies (e.g., NCT04092270). 

## 2. Results

### 2.1. Peposertib Exhibits Synergistic Antiproliferative Activity with TOPO II Inhibitors In Vitro

**Figure 1:**
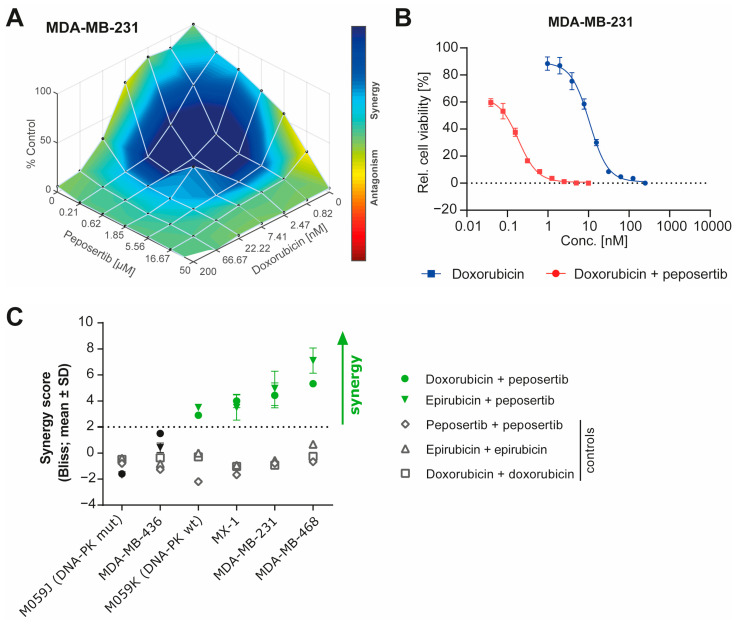
Peposertib synergistically enhances the cytotoxicity of doxorubicin in TNBC cell lines. (**A**) Overlay of Loewe synergy scores in MDA-MB-231 cell line after combination treatment of doxorubicin and peposertib at 168 h post treatment. Graphs were generated with Combenefit software version 2.021 using the cell viability data at 168 h. (**B**) Potentiation of doxorubicin cytotoxicity by 1 µM peposertib on MDA-MB-231 cells, as measured using an Resazurin/Alamar Blue viability assay 168 h post treatment. (**C**) Bliss synergy scores for peposertib with doxorubicin or epirubicin were assessed in a panel of breast cancer cell lines using combination dose matrices. A synergy score of >2 is indicative of synergism, and black data points indicate absence of synergy. M059J and M059K glioblastoma cell lines served as controls.

To assess whether the inhibition of DNA-PK using peposertib could potentiate the antiproliferative activity of Topo II inhibitors such as doxorubicin or etoposide, the MDA-MB-231 cell line was selected as a well-established TNBC model. Combination matrices involving multiple doses provided evidence that simultaneous administration of peposertib with doxorubicin (Figure 1A) or etoposide (Supplemental Figure S1A) confirmed the presence of synergistic treatment effects. In a subsequent study, we treated MDA-MB-231 cells with increasing doses of doxorubicin in the absence or presence of 1µM peposertib for 7 days and observed a 57-fold shift in the half-maximal inhibitory concentration (IC_50_) (Figure 1B). A comparable combination effect and IC_50_ shift were also observed with etoposide, another clinical TOPO II inhibitor (Supplemental Figure S1B).

We extended our combination study to a range of TNBC cell lines, utilizing multi-dose combination matrices. Upon combination with doxorubicin or epirubicin, peposertib exhibited synergistic antiproliferative activity, defined as a BLISS score of >2, in three out of four TNBC cancer cell lines (Figure 1C). The human glioma cell lines M059K (DNA-PK proficient) and M059J (DNA-PK deficient) were used as controls to demonstrate that the observed combination effects are mediated via selective DNA-PK inhibition [11].

### 2.2. Peposertib Synergizes with Doxorubicin to Promote TP53-Mediated G2/M Cell Cycle Arrest and Apoptosis

**Figure 2:**
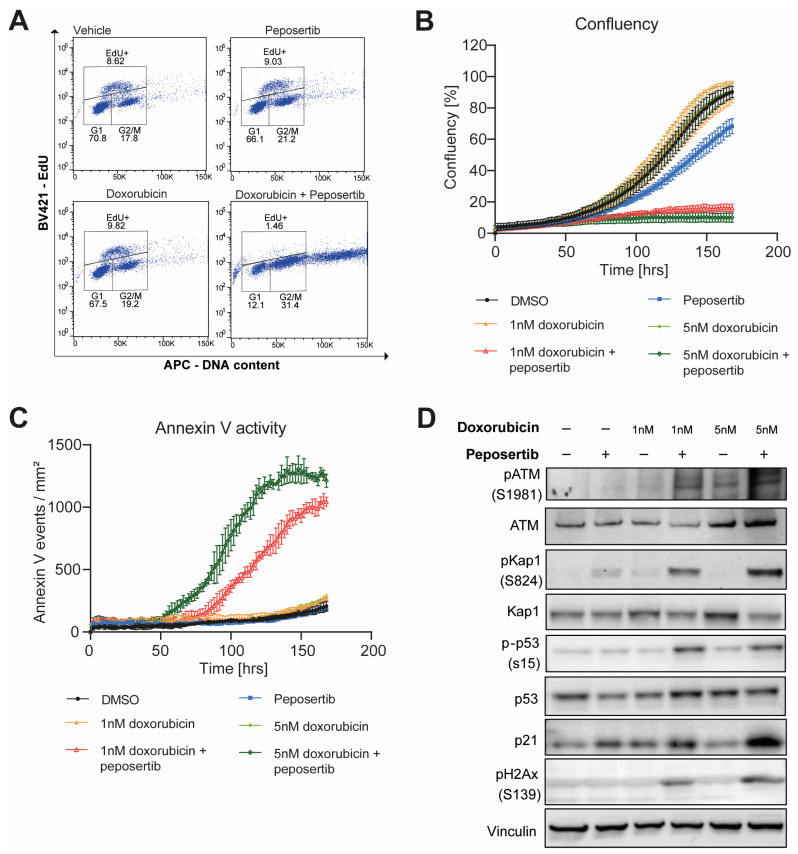
Combined treatment of peposertib and doxorubicin induces cell cycle arrest and apoptosis in MDA-MB-231 cells. (**A**) MDA-MB-231 cells were exposed to doxorubicin, peposertib, or their combination for a period of 7 days, and subjected to EdU proliferation and cell cycle analyses. Quantification of MDA-MB-231 (**B**) confluency and (**C**) Annexin V-Red positive cells over the course of treatment as monitored using the Incucyte^®^. (**D**) Cells were exposed to doxorubicin, peposertib or their combination for 7 days. Protein lysates were analyzed by Western blotting using antibodies against phospho-ATM(S1981), ATM, phospho-Kap1(S824), Kap1, phospho-p53(S15), p53, p21, phospho-gH2Ax(S139), and Vinculin (loading control). Enhanced ATM activity was evaluated as upregulation of phospho-ATM (S1981) itself and its direct phosphorylation targets p-Kap1 (Ser824) and p-p53 (Ser15). Accumulation of DNA damage is indicated by an increase in p-gH2AX in the combination groups.

To elucidate the potential mechanisms underlying the chemo-potentiation effect in MDA-MB-231 cells, we first performed an EdU proliferation assay and cell cycle analysis via flow cytometry. Compared to the vehicle group (8.62%), a single-agent treatment with 1µM peposertib or 1 nM doxorubicin in MDA-MB-231 cells did not affect the percentage of Edu+ cells in the culture (9.03% and 9.82%, respectively). However, the combination of both agents resulted in a significant reduction in EdU+ cells (1.46%), indicating reduced DNA replication with the combination treatment (Figure 2A). Cell cycle distribution was not affected by individual treatment with peposertib or doxorubicin, but upon combined treatment, an accumulation of cells in the G2/M phase was observed (Figure 2A). This suggests that cell cycle arrest occurred, possibly due to the presence of unrepaired DNA damage.

To investigate the fate of MDA-MB-231 cells exposed to combined treatment with peposertib and doxorubicin, we performed real-time analysis of cell growth and apoptosis using Incucyte^®^ Annexin V Red staining for a duration of 168 h. During this live cell imaging period, apoptotic cells were identified by the presence of red fluorescence staining. A qualitative assessment of bright-field images combined with Annexin V Red staining revealed a noticeable reduction in cell density at 72 h following co-treatment of peposertib and doxorubicin, as compared to the single-agent treatment (Supplemental Figure S2).

Furthermore, treatment with 1 µM peposertib or doxorubicin at 1 nm or 5 nm alone had no effect on apoptosis induction or cell growth. However, the combination of both drugs strongly increased the number of annexin V-positive events and inhibited cell growth over the course of the experiment (Figure 2B,C).

In a previous study involving leukemia cells, we showed that in the presence of DSB damage, peposertib disrupts a negative regulatory loop between DNA-PK and ATM. This disruption results in the enhanced activation of the ATM pathway, including its downstream target p53 [12]. Consequently, in cancer cells with functional p53, inhibiting DNA-PK amplifies the p53 response to DNA DSBs, leading to cell cycle arrest and apoptosis [12].

To investigate if a similar mechanism is also present in TNBC cells, we analyzed protein lysates generated from MDA-MB-231 cells treated with DMSO, peposertib, doxorubicin or the combination for 7 days. As depicted in Figure 2D, ATM undergoes autophosphorylation at serine 1981, indicating activation in response to the combination treatment. This activation is potentially a consequence of DNA DSB accumulation, as demonstrated by the induction of phospho-H2AX at Ser139. The phosphorylation of H2AX at Ser139, often referred to as γ-H2AX, is induced in response to DSBs originating from diverse cell intrinsic or external sources, and it serves as an established marker for the detection and quantification of DNA DSBs [13]. While treatment with doxorubicin and peposertib as single agents for 7 days had no effect on γ-H2AX levels in MDA-MB-231 cells, combined treatment led to a pronounced increase, indicating an accumulation of DNA DSBs. Furthermore, ATM activation in the combination treatment was also manifested through the upregulation of its direct phosphorylation targets p-Kap1 (Ser824) and p-p53 (Ser15), as well as in the upregulation of the p53 transcriptional target p21, which serves as a key driver of p53-dependent cell cycle arrest and apoptosis, respectively (Figure 2D).

### 2.3. Combined Treatment of Peposertib with Pegylated Liposomal Doxorubicin (PLD) Suppresses Tumor Growth

Next, we investigated if peposertib could enhance the anti-tumor efficacy of PLD in in vivo xenograft studies. We selected two TNBC xenograft models, namely MDA-MB-231 and MX-1, and used the treatment-to-control ratio ΔT/ΔC (also referred to as T/C or % T/C) to quantify the anti-tumor activity of treatments. We also analyzed changes in body weight as a surrogate parameter of tolerability. Mice harboring established subcutaneous tumors were randomized into four treatment groups (n = 10 each): a vehicle control group, intravenously administered PLD monotherapy once weekly (QW) at a dose of 3 mg/kg; oral peposertib monotherapy administered bi-daily (BID) at a dose of 100mg/kg, four days per week; or the combined treatment of PLD monotherapy on day 1 followed by four days of BID treatment with peposertib at 100 mg/kg from days 2–5, with a two-day treatment pause. A visual representation of the applied weekly combination treatment schedule is shown in Supplemental Figure S3A.

**Figure 3:**
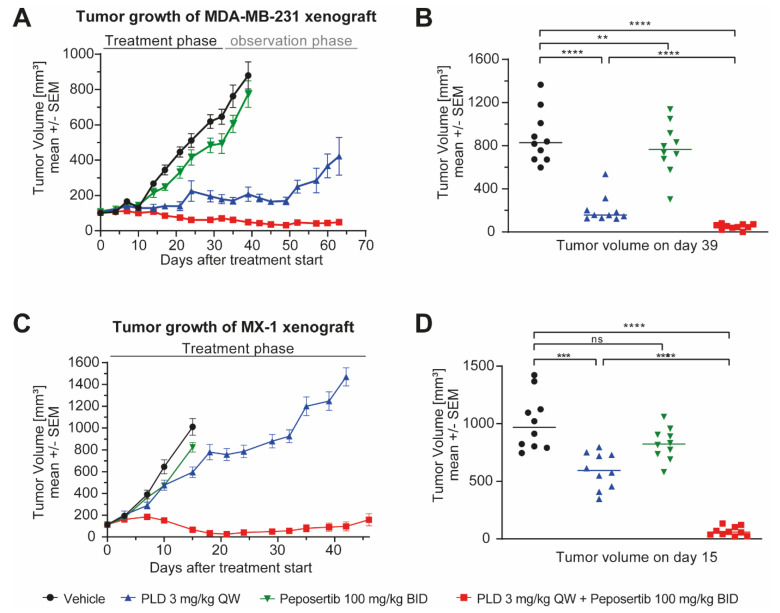
Peposertib enhances the anti-tumor activity of PLD in subcutaneous xenograft tumors. Tumor growth of (**A**) MDA-MB-231 and (**C**) MX-1 xenografts treated with vehicle control, peposertib, PLD or combinations thereof (n = 10 for all groups, mean ± SEM). Median tumor volumes of (**B**) MDA-MB-231 and (**D**) MX-1 on day 39 and day 15, respectively. Statistical analysis was performed using two-way ANOVA. ** *p*-value = 0.0076, *** *p*-value = 0.0006, **** *p*-value < 0.0001, ns, not significant.

In MDA-MB-231 tumor-bearing animals, peposertib exhibited moderate but statistically significant anti-tumor activity (T/C = 85.3%). PLD in monotherapy had pronounced anti-tumor activity (T/C = 13.6%) but was not able to induce tumor regressions during the course of treatment, and tumors began regrowing upon treatment cessation. However, the combined treatment of PLD and peposertib was able to induce tumor regression (T/C = −7.6%) and prevented tumor regrowth during the observation period, indicating the durable anti-tumor effects of this combination (Figure 3A,B). None of the treatments resulted in body weight loss in the animals, indicating good tolerability of the combination treatment (Supplemental Figure S3B). In MX-1 xenografts, treatment with peposertib monotherapy showed no significant effect on tumor growth (T/C = 76.2%), while PLD monotherapy exhibited a moderate but statistically significant tumor growth inhibition effect (T/C = 53.6%). The combination of PLD and peposertib outperformed all other treatments and induced long-lasting tumor regression (T/C = −5.3%) in most of the animals (Figure 3C,D). Also, no body weight loss was observed throughout the study (Supplemental Figure S3C). In addition, to evaluate the antiproliferative activity of the combination treatment, immunohistochemical analysis of Ki67 was performed on MDA-MB-231 tumors excised from mice sacrificed on day 39. At least three mice were sacrificed from each treatment group, and the tumors were stained and quantified for Ki67. Ki67 staining was significantly reduced in the PLD + peposertib group in comparison to vehicle or single-agent treatment (*p*-value < 0.0001; Supplemental Figure S4). In summary, these data demonstrate that the combination of PLD + peposertib is well tolerated and achieves superior anti-tumor efficacy compared to single-agent treatments.

**Figure 4:**
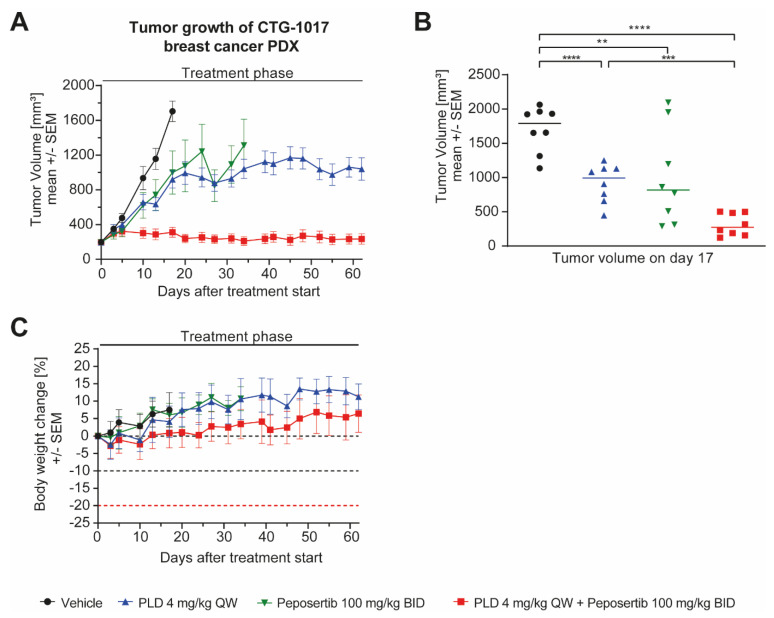
Peposertib enhances the anti-tumor activity of PLD in a human TNBC PDX model. (**A**) Tumor growth of CTG-1017 TNBC PDX xenografts treated with vehicle control, peposertib, PLD or combinations thereof (n = 8 for all groups, mean ± SEM). (**B**) Median tumor volume on day 17 and (**C**) corresponding mouse bodyweight change over time. Statistical analysis was performed using two-way ANOVA. ** *p*-value = 0.0073, *** *p*-value = 0.0005, **** *p*-value < 0.0001.

Next, we evaluated the anti-tumor efficacy of the combination in the patient-derived xenograft (PDX) model CTG-1017 (Champions Oncology, Rockland, MA, USA). This model was derived from a 56-year-old patient diagnosed with stage 3 invasive non-metastatic triple-negative ductal carcinoma of the breast. To evaluate the combination potential of PLD with peposertib in this PDX model, mice harboring established subcutaneous tumors were randomized into four treatment groups and treated with either vehicle control, PLD monotherapy, peposertib monotherapy or the combination of PLD and peposertib using the same schedule as described above. Moderate but statistically significant tumor growth inhibition effects were observed for peposertib (T/C = 53.2%) or PLD (T/C = 48.0%) monotherapy, but the tumors continued to grow during the course of the experiment. In contrast, the combined treatment of PLD and peposertib effectively blocked tumor growth throughout the study (T/C = 7.7%) and achieved statistically superior anti-tumor efficacy compared to single-agent treatments (Figure 4A,B). Unlike our studies in cell-line-derived xenograft models, we used a dose of 4mg/kg PLD in the monotherapy and combination treatments, but the tolerability of the combined treatment remained excellent based on body weight changes and the absence of any clinical symptoms during the study.

## 3. Discussion

Our study presents compelling evidence that a notable enhancement in TOPO II inhibitor-mediated cytotoxicity can be achieved in preclinical TNBC models through combination with peposertib, a highly selective and potent DNA-PK inhibitor. This discovery is significant because the current standard treatment for TNBC relies on anthracyclines like epirubicin, which exert cytotoxic effects by inhibiting TOPO II. The utilization and effectiveness of anthracyclines such as epirubicin or doxorubicin are often constrained because of their associated side effects, such as cardiotoxicity [14,15]. Moreover, TNBC presents a challenging clinical scenario, characterized by a scarcity of actionable molecular targets, leaving chemotherapy as the preferred treatment of choice, with all its advantages and limitations [16]. 

The results observed in our in vitro studies indicate that peposertib synergistically enhances the cytotoxicity of TOPO II inhibitors, such as doxorubicin, epirubicin and etoposide (Figure 1 and Supplemental Figure S1). This enhancement can be attributed to the complementary mechanisms between the induction of DNA damage by the TOPO II inhibitor and the inhibition of relevant DNA-PK-mediated repair mechanisms by peposertib. Our studies demonstrate that the increased induction of DNA damage was associated with replication arrest and aneuploidy, probably leading to cell death through apoptosis in vitro (Figure 2). Importantly, ATM activation plays a pivotal role in this process, as it senses the DNA damage caused by the combination treatment and becomes activated, as shown in Figure 2D. While ATM activation in normal cells aids in the repair of DNA damage, regulation of cell cycle checkpoints and maintenance of genomic stability, excessive DNA damage can shift the balance of ATM activation towards apoptosis induction [17]. It is known that ATM is involved in the DNA damage response-induced activation of the intrinsic apoptotic pathway, predominantly through a p53-mediated mechanism [18] (Figure 2D). ATM and its downstream effectors phosphorylate p53, leading to its stabilization and the induction of various pro-apoptotic genes, thus facilitating the mitochondrial apoptotic pathway [19].

We established a well-tolerated treatment regimen for the combined application of peposertib and PLD in vivo, and our studies in mice harboring subcutaneously implanted tumors demonstrated a remarkable enhancement in anti-tumor efficacy when peposertib and PLD were co-administered (Figure 3 and Figure 4). The combined treatment resulted in a significant and long-lasting delay in tumor growth. Importantly, the combination therapy exhibited minimal observable toxicity, indicating its potential as a well-tolerated treatment option. This is of the utmost significance as minimizing treatment-related toxicity is crucial for improving patient outcomes. Furthermore, our study yielded promising results in tumor models with inherent resistance to chemotherapy treatment. We observed that the combination therapy effectively circumvented the intrinsic resistance mechanisms and demonstrated potent anti-tumor activity in tumor models that did not respond to TOPO II inhibitor treatment alone (MX-1 and CTG-1017). 

The CTG-1017 PDX model (Figure 4) was derived from a patient diagnosed with a stage 3 invasive non-metastatic triple-negative ductal carcinoma of the breast. Important in the context of this manuscript, the patient had previously received two lines of chemotherapy (epirubicin + cyclophosphamide and docetaxel + carboplatin) before tumor material was resected for PDX establishment. However, the patient did not show any objective response to these treatments, indicating intrinsic resistance to TOPO II inhibitors and other commonly used chemotherapies. Molecular characterization of the established PDX model revealed a low mutational burden and no mutations in tumor suppressor genes, such as *BRCA1*, *BRCA2*, *TP53* or *RB1*. Additionally, no mutations were identified in oncogenes, like *PIK3CA*, *KRAS* or *CDK6*, which, overall, provides no clear scientific rationale for any targeted therapy approach. Our study confirmed TOPO II inhibitor resistance, as evidenced by the lack of significant efficacy of PLD monotherapy at the tested dose. However, the combination with peposertib successfully surmounted treatment resistance (Figure 4). 

In the context of DNA-PK inhibitors, alongside peposertib, other compounds such as AZD7648 have been developed for clinical testing. Our study’s findings are consistent with previously published data on AZD7648, which also achieved synergistic anti-tumor effects when combined with PLD in preclinical ovarian and breast cancer models [20,21]. While the comparable outcomes in this study support the potential benefits of combining DNA-PK inhibitors with TOPO II inhibitor-based therapies, our study adds further insights to the underlying mechanism. Specifically, our findings reveal that the combined treatment of PLD and peposertib induces apoptosis by activating the ATM pathway. By elucidating this mechanism of action, our study enhances the understanding of how combination therapy exerts its anti-tumor effects. Additionally, our findings emphasize the ability of peposertib to overcome resistance to TOPO II inhibitor-based treatments. This finding distinguishes our study from previous investigations involving other DNA-PK inhibitors, which did not report details on the underlying MoA and the possibility to overcome TOPO II inhibitor resistance in TNBC models. Noteworthily, the effectiveness of the combination of PLD and peposertib extends beyond TNBC. Recently published studies have also demonstrated the efficacy of the combination in preclinical ovarian cancer models [22] and sarcoma models [23,24], highlighting the versatility of this combination therapy across multiple tumor types. These findings expand the opportunities for clinical explorations and suggest a potentially broader scope of application for the PLD and peposertib combination beyond the specific tumor types studied.

Apart from investigations on peposertib in clinical studies, there are no ongoing trials specifically studying the combination of other DNA-PK inhibitors with TOPO II inhibitors. However, there are several ongoing clinical studies focusing on peposertib, including two trials (NCT04092270 and NCT05711615) examining the combination of PLD and peposertib for the treatment of ovarian cancer and leiomyosarcoma. These trials underscore the increasing interest in investigating this combination therapy as a potential treatment option.

In summary, our study provides insights into the effectiveness and underlying mechanisms of combining PLD and peposertib. We demonstrate that this combination induces apoptosis through the activation of the ATM pathway and can reverse resistance to TOPO II inhibitors. These findings emphasize the clinical value of combining PLD and peposertib to enhance the therapeutic outcomes of anthracycline-based and TOPO II inhibitor-based regimens. Further research and clinical investigations are necessary to validate these preclinical results and translate them into clinical practice, benefiting patients with TNBC.

## 4. Material and Methods

### 4.1. Reagents and Cell Culture 

Peposertib was synthesized at Merck Healthcare KGaA, Darmstadt, Germany. Doxorubicin and etoposide were purchased from Sigma-Aldrich (St. Louis, MO, USA). For in vitro experiments, drugs were solubilized in DMSO to create stock solutions, which were then frozen and stored at −20 °C until needed. The concentration of DMSO in the media did not exceed 0.1% (*vol*/*vol*). 

MDA-MB-231 cell line was purchased from ATCC and was cultured in Dulbecco’s Modified Eagle Medium (DMEM) + 10% fetal calf serum at 37 °C with 10% CO_2_. MX-1 cell line was purchased from NCI-Frederic Cancer DCT tumor repository and was cultured in DMEM/F12 medium supplemented with 7.5% FBS and 2mM L-Glutamine at 37 °C with 5% CO_2_. All other cell lines used in this study are listed in Supplementary Table S1. Cell line identity was confirmed by short tandem repeat (STR) analyses; mycoplasma and bacterial contamination was excluded.

### 4.2. Cell Viability Assay

For viability and combination matrix assays, MDA-MB-231 cells were plated at 1000 cells per well in 96-well plates. The next day, cells were treated with serial dilution of drugs using a Tecan D300e Digital Despenser, and DMSO concentration was normalized in all wells. At 168 h (7 days) following the drug treatment, effect on cell growth or viability was assessed with Resazurin assay according to manufacturer’s protocol, and fluorescent signal was recorded using a Tecan Connect plate reader. 

Dose response curve and IC_50_ values were generated using Graphpad Prism (v9.0.0), while for the combination matrix, synergism was analyzed with Loewe’s additivity or Bliss independent model using GeneData Screener Software (Version 19.0.5); Loewe’s model was graphed using Combenefit software (v.2.021) [25].

### 4.3. Immunoblotting 

Cells were harvested and lysed in RIPA buffer, supplemented with both protease and phosphatase inhibitors (Roche Diagnostics, Rotkreuz, Switzerland): 20 mM Tris-HCl (pH 7.5), 150 mM NaCl, 1 mM Na2EDTA, 1 mM EGTA, 1% NP-40, 1% sodium deoxycholate, 2.5 mM sodium pyrophosphate, 1 mM beta-glycerophosphate, 1 mM Na3VO4, 1 µg/mL leupeptin (Cell Signaling Technology, Danvers, MA, USA). To ensure lysis, cells were sonicated with Diagenode Bioruptor Plus for 10 min (30 s on/30 s off cycle) at 4 °C and subsequently centrifuged at 4 °C at 13,000 rpm. Protein concentration was determined by the BCA protein assay (Thermo Fisher Scientific, Waltham, MA, USA), and equal amount of protein was mixed with 4× NuPAGE LDS Sample Buffer (Invitrogen, Waltham, MA, USA) and 10× NuPAGE Reducing Agent (Invitrogen). Samples were heated at 70 °C for 10 min prior to separation on NuPAGE 4–12% BisTris Mini Protein gels (Invitrogen) and NuPAGE MOPS SDS Running Buffer (Invitrogen). Proteins were subsequently transferred to polyvinylidene fluoride (PVDF) membrane via iBlot Dry Blotting System (Thermo Fisher Scientific). Membranes were incubated with the appropriate antibodies and imaged with Bio-rad ChemiDoc Imaging System using Western Lighting Plus ECL (PerkinElmer, Waltham, MA, USA). A list of the antibodies and their sources can be found in Supplementary Table S2. 

### 4.4. IncuCyte Live Cell Imaging 

MDA-MB-231 cells were plated in 96-well plates and incubated overnight before drugs, and IncuCyte Annexin V red reagent (Essen Bioscience, Ann Arbor, MI, USA) was added the next day to label apoptotic cells in real time. Cells were imaged using 10× objective in Incucyte S3 device at 2 h intervals for 7 days. Relative apoptosis events were determined by the number of Annexin V red counts per mm^2^ normalized to percent confluence.

### 4.5. Flow Cytometry Analysis

Cells were treated with doxorubicin, peposertib or the combination of doxorubicin and peposertib for 7 days (168 h) and labelled with EdU using Click-iT™ Plus EdU Pacific Blue™ flow cytometry assay kit (Thermo Fisher Scientific, Waltham, MA, USA) according to the manufacturer’s protocol. Briefly, cells were pulsed with 10 μM EdU for 1 h and fixed with supplied Click-iT™ fixative for 10 min at RT. Afterwards, cells were washed once with 1% BSA in PBS and permeabilized with Click-iT™ permeabilization and wash reagent at RT for 10 min. Click-iT™ Plus reaction cocktail was then added directly to the fixed and permeabilized cells and incubated for 30 min at RT. Then, cells were washed and incubated with SYTO™ Deep Red Nucleic Acid Stain (Thermo Fisher Scientific, Waltham, MA, USA) for DNA content staining at 37 °C for 1 h. Cells were analyzed by BD FACS Celesta flow cytometer, and data were processed with FlowJo software (v10.7.1).

### 4.6. Immunohistochemistry 

Samples were fixed in 4% paraformaldehyde for 36–48 hours at room temperature, then dehydrated and embedded in paraffin. Using a Microtome Leica RM 2255 (Leica Microsystems, Tokyo, Japan), they were sliced into 3–4 µm thick sections and mounted onto Matsunami TOMO^®^ hydrophilic adhesion slides for immunohistochemistry (IHC) staining.

The staining procedure starting with the deparaffinization of sections was performed with the staining instrument Discovery^®^ Ultra (Ventana Medical Systems, Inc. (VSMI), Tucson, AZ, USA). After deparaffinization, sections were heated for epitope retrieval in Tris-EDTA buffer pH 8.4. Endogenous peroxidase was blocked by incubation in 3% hydrogen peroxide (part of detection kit, Roche). Sections were incubated with antibody diluent (Roche) diluted primary and secondary antibodies (Supplemental Table S2). Finally, the horseradish peroxidase (HRP)-conjugated polymers of the detection kit (OmniMap anti-rabbit HRP from VSMI) were used to catalyze the 3,3′-diaminobenzidine tetrahydorchloride (DAB)/H_2_O_2_ reaction (ChromoMap, VSMI) to produce an insoluble dark-brown precipitate that can be visualized. Sections were counterstained with hematoxylin (Hematoxylin II, VSMI). Slides were washed in tap water, dehydrated, and mounted with glass coverslips in permanent mounting media Entellan^®^ Neu (VWR, Darmstadt, Germany).

Immunohistochemical stains were then scanned with the Hamamatsu NanoZommer S210. The scans were analyzed with the image analysis software HALO v3.6 (Indica Labs, Albuquerque, NM, USA). Ki67 positive cells were quantified using the cytonuclear detection algorithm v2.0.9 in viable tumor regions that were annotated by hand. Data were exported to Excel and visualized in GraphPad Prism v.9.1.2.

### 4.7. Animal Studies 

In vivo efficacy data were generated in subcutaneous human cell-line derived xenograft and patient-derived xenograft (PDX) models. For human cell-line derived xenograft tumors, 10 million MDA-MB-231 cells were injected subcutaneously (s.c.) in 1:1 (v:v) DPBS/Matrigel Basement Membrane Matrix near the mammary fat pad of female 8–10 weeks old H2d Rag2 mice [C;129P2-H2^d^-TgH(II2rg)^tm1Brn^-TgH(Rag2)^tm1Alt^N4] (Taconic Biosciences, Lille Skensved, Dänemark). For MX-1 xenograft model, 5 million cells were injected s.c. in DPBS near the mammary fat pad of 8–10 weeks old female CIEA-BRG mice [C.Cg-Rag2^tm1Fwa^ Il2rg^tm1Sug^/JicTac] (Taconic Biosciences, Lille Skensved, Dänemark). Study was randomized into groups (N = 10/group) of equal mean tumor volume (TV) prior to treatment. All studies were approved by the local animal welfare authority (Regierungspräsidium Darmstadt, Hesse, Germany; experimental license number DA4/Anz.1040). 

The efficacy study in the TNBC PDX models CTG-1017 was performed at Champions Oncology according to the guidelines of the Institutional Animal Care and Use Committee (IACUC) of Champions Oncology. Athymic Nude-Foxn1nu (Immune-compromised) mice were implanted with fragment from Champions TumorGraft^®^ models CTG-1017. After tumors reached 1000–1500 mm^3^, they were harvested, and tumor fragments were implanted s.c. in the left flank of female study mice. Tumor growth was monitored twice a week using digital calipers, and TV was calculated using the formula (0.52 × [length × width^2^]). When TV reached approximately 150–300 mm^3^, animals were matched by tumor size and assigned into vehicle control or treatment groups (n = 8/group), and dosing was initiated on d0 up to d62 or until mean TV in one group reached 1500 mm^3^. Tumor size and body weight was measured twice a week. Histopathological and molecular analyses were performed at Champions Oncology and data was reviewed at Merck Healthcare KGaA.

For all in vivo studies peposertib was formulated in vehicle (0.5% Methocel, 0.25% Tween20, 300 mmol/L sodium citrate buffer, pH 2.5 and administered orally. Doxorubicin or pegylated liposomal doxorubicin formulated for intravenous administration in 5% (50 mg/mL) glucose solution was injected into the tail vein once weekly at the indicated dose.

### 4.8. Ethical Approval

All animal experiments were performed at either Merck Healthcare KGaA, Darmstadt, Germany or Champion Oncology and were carried out in accordance with relevant guidelines and regulations. This study is conducted and reported in accordance with ARRIVE guidelines.

## Data Availability

Any requests for data by qualified scientific and medical researchers for legitimate research purposes will be subject to Merck’s (CrossRef Funder ID: 10.13039/100009945) Data Sharing Policy. All requests should be submitted in writing to Merck’s data sharing portal (https://www.merckgroup.com/en/research/our-approach-to-research-and-development/healthcare/clinical-trials/commitment-responsible-data-sharing.html). When Merck has a co-research, co-development, or co-marketing or co-promotion agreement, or when the product has been out-licensed, the responsibility for disclosure might be dependent on the agreement between parties. Under these circumstances, Merck will endeavor to gain agreement to share data in response to requests.

## Supplementary Materials

The following supporting information can be downloaded at: https://www.mdpi.com/article/10.3390/ijms25105120/s1.

## References

[1] Wu Q., Siddharth S., Sharma D. (2021). Triple Negative Breast Cancer: A Mountain Yet to Be Scaled Despite the Triumphs. Cancers.

[2] Dent R., Trudeau M., Pritchard K.I., Hanna W.M., Kahn H.K., Sawka C.A., Lickley L.A., Rawlinson E., Sun P., Narod S.A. (2007). Triple-negative breast cancer: Clinical features and patterns of recurrence. Clin. Cancer Res..

[3] Davison C., Morelli R., Knowlson C., McKechnie M., Carson R., Stachtea X., McLaughlin K.A., Prise V.E., Savage K., Wilson R.H. (2021). Targeting nucleotide metabolism enhances the efficacy of anthracyclines and anti-metabolites in triple-negative breast cancer. npj Breast Cancer.

[4] Bianchini G., Balko J.M., Mayer I.A., Sanders M.E., Gianni L. (2016). Triple-negative breast cancer: Challenges and opportunities of a heterogeneous disease. Nat. Rev. Clin. Oncol..

[5] Nitiss J.L. (2009). Targeting DNA topoisomerase II in cancer chemotherapy. Nat. Rev. Cancer.

[6] Malik M., Nitiss K.C., Enriquez-Rios V., Nitiss J.L. (2006). Roles of nonhomologous end-joining pathways in surviving topoisomerase II-mediated DNA damage. Mol. Cancer Ther..

[7] Wang J.C. (2002). Cellular roles of DNA topoisomerases: A molecular perspective. Nat. Rev. Mol. Cell Biol..

[8] Zenke F.T., Zimmermann A., Sirrenberg C., Dahmen H., Kirkin V., Pehl U., Grombacher T., Wilm C., Fuchss T., Amendt C. (2020). Pharmacologic Inhibitor of DNA-PK, M3814, Potentiates Radiotherapy and Regresses Human Tumors in Mouse Models. Mol. Cancer Ther..

[9] Becker A., Krebs-Brown A., Vetter C., Reuter T., Rodriguez-Gutierrez A., You X., Lissy M. (2023). Phase I crossover study of DNA-protein kinase inhibitor peposertib in healthy volunteers: Effect of food and pharmacokinetics of an oral suspension. Clin. Transl. Sci..

[10] Samuels M., Falkenius J., Bar-Ad V., Dunst J., van Triest B., Yachnin J., Rodriguez-Gutierrez A., Kuipers M., You X., Sarholz B. (2023). A Phase 1 Study of the DNA-PK Inhibitor Peposertib in Combination with Radiation Therapy with or without Cisplatin in Patients with Advanced Head and Neck Tumors. Int. J. Radiat. Oncol. Biol. Phys..

[11] Allalunis-Turner M.J., Barron G.M., Day R.S., Dobler K.D., Mirzayans R. (1993). Isolation of two cell lines from a human malignant glioma specimen differing in sensitivity to radiation and chemotherapeutic drugs. Radiat. Res..

[12] Haines E., Nishida Y., Carr M.I., Montoya R.H., Ostermann L.B., Zhang W., Zenke F.T., Blaukat A., Andreeff M., Vassilev L.T. (2021). DNA-PK inhibitor peposertib enhances p53-dependent cytotoxicity of DNA double-strand break inducing therapy in acute leukemia. Sci. Rep..

[13] Fernandez-Capetillo O., Lee A., Nussenzweig M., Nussenzweig A. (2004). H2AX: The histone guardian of the genome. DNA Repair.

[14] Chlebowski R.T. (1979). Adriamycin (doxorubicin) cardiotoxicity: A review. West. J. Med..

[15] Furlanetto J., Loibl S. (2020). Optimal Systemic Treatment for Early Triple-Negative Breast Cancer. Breast Care.

[16] Obidiro O., Battogtokh G., Akala E.O. (2023). Triple Negative Breast Cancer Treatment Options and Limitations: Future Outlook. Pharmaceutics.

[17] Aki T., Uemura K. (2021). Cell Death and Survival Pathways Involving ATM Protein Kinase. Genes.

[18] Enoch T., Norbury C. (1995). Cellular responses to DNA damage: Cell-cycle checkpoints, apoptosis and the roles of p53 and ATM. Trends Biochem. Sci..

[19] Miyashita T., Reed J.C. (1995). Tumor suppressor p53 is a direct transcriptional activator of the human bax gene. Cell.

[20] Fok J.H.L., Ramos-Montoya A., Vazquez-Chantada M., Wijnhoven P.W.G., Follia V., James N., Farrington P.M., Karmokar A., Willis S.E., Cairns J. (2019). AZD7648 is a potent and selective DNA-PK inhibitor that enhances radiation, chemotherapy and olaparib activity. Nat. Commun..

[21] Anastasia A., Dellavedova G., Ramos-Montoya A., James N., Chiorino G., Russo M., Baakza H., Wilson J., Ghilardi C., Cadogan E.B. (2022). The DNA-PK Inhibitor AZD7648 Sensitizes Patient-Derived Ovarian Cancer Xenografts to Pegylated Liposomal Doxorubicin and Olaparib Preventing Abdominal Metastases. Mol. Cancer Ther..

[22] Wise H.C., Iyer G.V., Moore K., Temkin S.M., Gordon S., Aghajanian C., Grisham R.N. (2019). Activity of M3814, an Oral DNA-PK Inhibitor, In Combination with Topoisomerase II Inhibitors in Ovarian Cancer Models. Sci. Rep..

[23] Mariño-Enríquez A., Novotny J.P., Gulhan D.C., Klooster I., Tran A.V., Kasbo M., Lundberg M.Z., Ou W.B., Tao D.L., Pilco-Janeta D.F. (2023). Hyper-Dependence on NHEJ Enables Synergy Between DNA-PK Inhibitors and Low-Dose Doxorubicin in Leiomyosarcoma. Clin. Cancer Res..

[24] Revia S., Budzinska M.A., Bogatyrova O., Neumann F., Zimmermann A., Amendt C., Albers J. (2023). DNA-Dependent Protein Kinase Inhibitor Peposertib Potentiates the Cytotoxicity of Topoisomerase II Inhibitors in Synovial Sarcoma Models. Cancers.

[25] Di Veroli G.Y., Fornari C., Wang D., Mollard S., Bramhall J.L., Richards F.M., Jodrell D.I. (2016). Combenefit: An interactive platform for the analysis and visualization of drug combinations. Bioinformatics.

