# Supplementary material for: Peposertib, a DNA-PK Inhibitor, Enhances the Anti-Tumor Efficacy of Topoisomerase II Inhibitors in Triple-Negative Breast Cancer Models
Source: Int J Mol Sci. 2024 May 8;25(10):5120. doi: 10.3390/ijms25105120 (PMC11121553; doi:10.3390/ijms25105120)
Supplement: Supplementary file 1 [file ijms-25-05120-s001.zip › ijms-2957209-supplementary.pdf]

Supplementary Figures and Tables

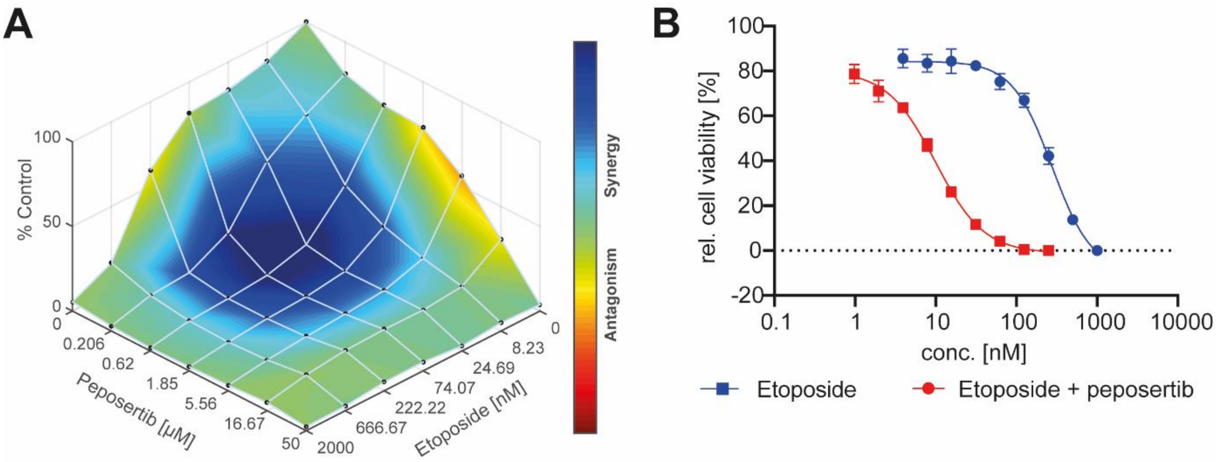

Suppl figure S1. Peposertib exhibits synergistic antiproliferative activity with etoposide. (A) Overlays of Bliss synergy matrices on combination dose response MDA-MB-231 cell line treated with etoposide and peposertib for 168 h. (B) Potentiation of etoposide cytotoxicity by 1 µM peposertib on MDA-MB-231 cells as measured using an Resazurin/ Alamar Blue viability assay at 168 h post treatment.

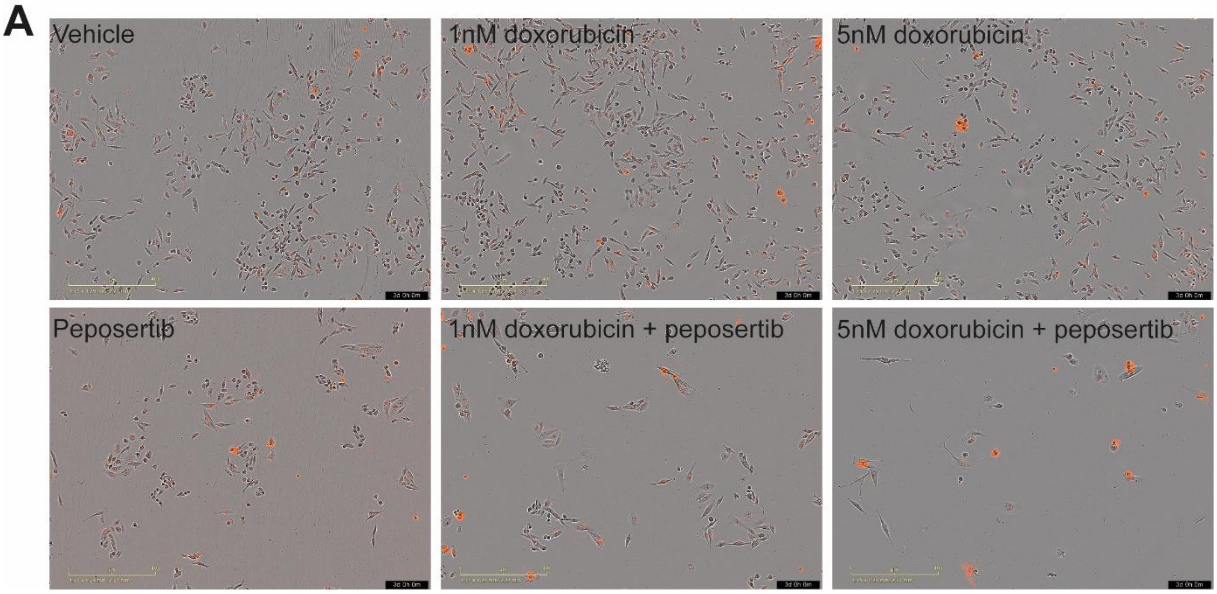

Suppl. figure S2. Peposertib, in combination with doxorubicin, affect cell proliferation and induce apoptosis in TNBC cell line. (A) Incucyte® bright-field images of MDA-MB-231 at 72 hours post treatment at 10x magnification overlay with AnnexinV-Red staining. Images are representative from 3 independent replicates.

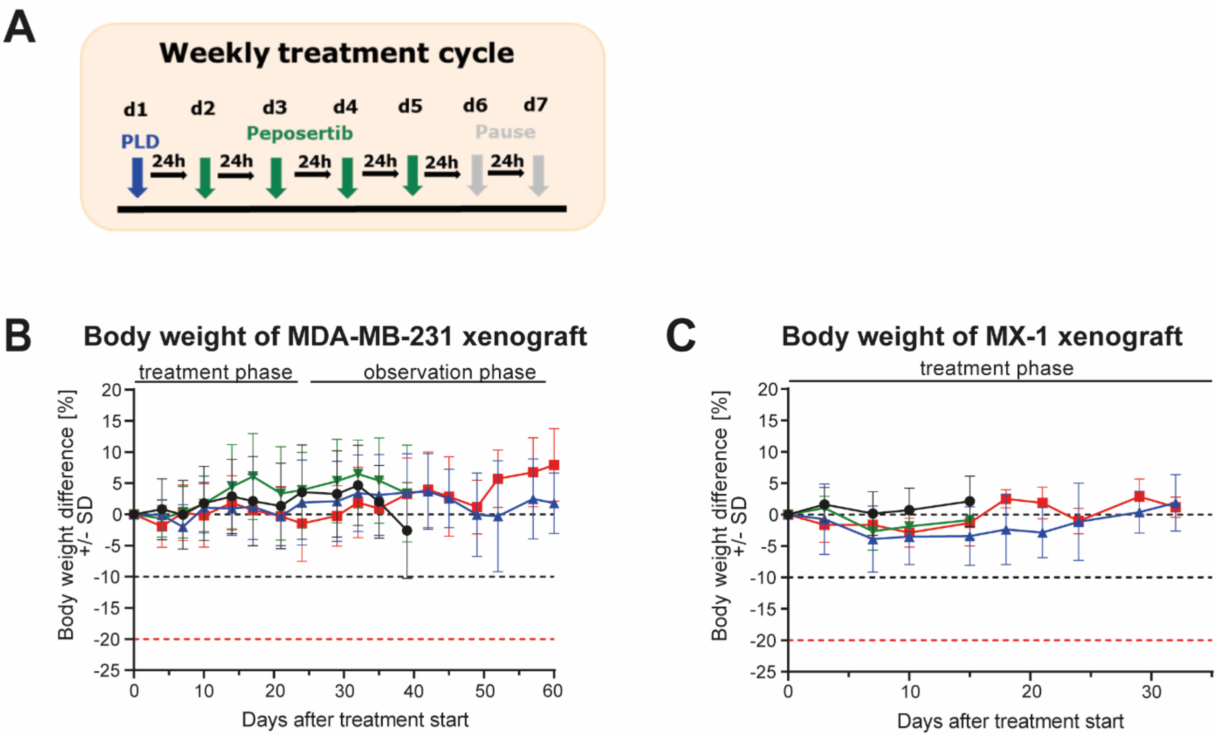

Suppl. figure S3. Combination treatment of peposertib with PLD in human TNBC xenograft models *in vivo* results in DNA damage accumulation and is well tolerated. (A) Western blot analysis of tumor lysates obtained from mice harboring MDA-MB-231 xenografts that were treated with PLD or PLD + peposertib. Unless, otherwise indicated, in the combination group, co-treatment with peposertib (twice daily) for four days was started 24h after PLD treatment. Lysates obtained from at least 3 tumors/treatment groups were pooled for this analysis. (B) Scheme of the treatment schedule used in the *in vivo* efficacy studies for PLD + peposertib. Relative body weight changes of (C) MDA-MB-231 and (D) MX-1 tumor-bearing mice throughout the studies.

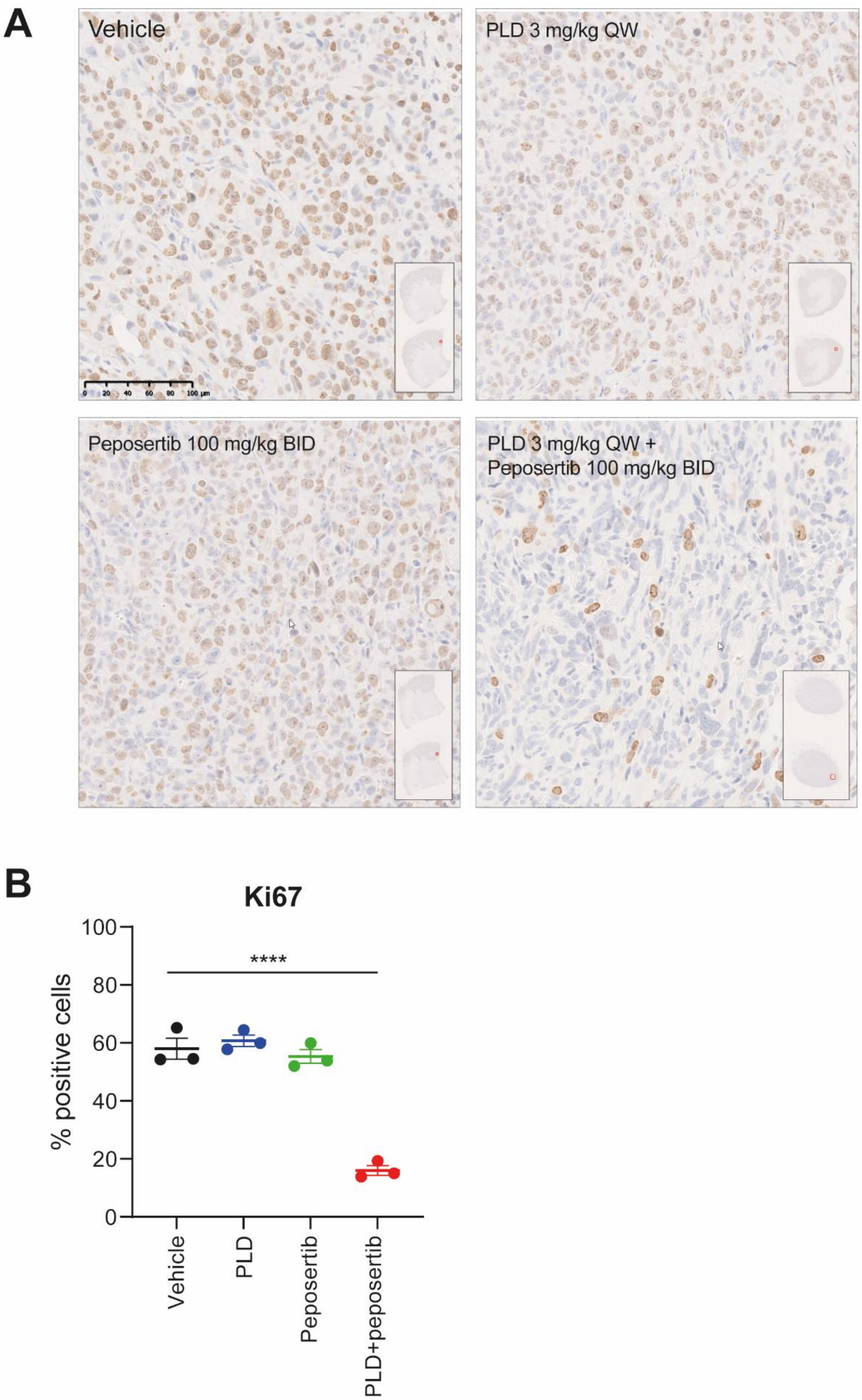

Suppl. figure S4 Combination treatment reduced Ki67-positive staining in cell-derived xenograft tumors. (A) Representative Ki67 immunohistochemical staining in MDA-MB-231 xenograft tumors at day 39 apost-treatment and (B) its quantification. Groups were compared by one-way ANOVA followed by Dunn's Multiple Comparison Test using one-way ANOVA.

Supplementary table S1

Cell line	Source	Culture condition
MDA-MB-231	ATCC	DMEM + 10% FBS
MDA-MB-436	ATCC	RPMI 1640 + 10% FBS + Sodium Pyruvate
MDA-MB-468	ATCC	RPMI 1640 + 10% FBS + Sodium Pyruvate + L-Glutamine
MX-1	DCTD Tumor Repository	DMEM/F12 + 7.5% FBS + L-Glutamine
M059J	ATCC	DMEM/F12 + 10% FBS + L-Glutamine + NEAA
M059K	ATCC	DMEM/F12 + 10% FBS + L-Glutamine + NEAA

Supplementary table S2

Antibodies	Usage (dilution)	Source	Identifier
p-H2Ax (S139)	WB (1:2000)	Sigma Millipore	05-636
p21	WB (1:1000)	Cell Signaling Technology	2947
p53	WB (1:500)	Santa Cruz	sc-126
p-p53(Ser15)	WB (1:1000)	Cell Signaling Technology	9284
Kap1	WB (1:1000)	Cell Signaling Technology	4124
pKap1 (S824)	WB (1:1000)	Abcam	ab133440
ATM	WB (1:1000)	Cell Signaling Technology	2873
pATM (S1981)	WB (1:1000)	Cell Signaling Technology	4526
Vinculin	WB (1:5000)	Sigma	V9131
Goat anti-Mouse IgG (H+L) Secondary Antibody, HRP	WB (1:5000)	Invitrogen	31430
Goat anti-Rabbit IgG (H+L) Secondary Antibody, HRP	WB (1:5000)	Invitrogen	31460
Ki67 (SP6)	IHC (1:500)	abcam	ab16667
OmniMap anti-rabbit HRP	IHC	Roche	760-4311
